# Estimating genomic instability mediated by *Alu* retroelements in breast cancer

**DOI:** 10.1590/S1415-47572009005000018

**Published:** 2009-01-23

**Authors:** Ana Cristina Fazza, Flavia Cal Sabino, Nathalia de Setta, Newton Antonio Bordin, Eloiza Helena Tajara da Silva, Claudia Marcia Aparecida Carareto

**Affiliations:** 1 Departamento de Biologia, Instituto de Biociências, Letras e Ciências Exatas, Universidade Estadual Paulista Júlio De Mesquita Filho, São José Rio Preto, SPBrazil; 2 Departamento de Ginecologia e Obstetrícia, Faculdade de Medicina, São José do Rio Preto, SPBrazil; 3 Departamento de Biologia Molecular, Faculdade de Medicina, São José do Rio Preto, SPBrazil; 4 Departamento de Genética e Biologia Evolucionária, Instituto de Biociências, Universidade de São Paulo, São Paulo, SPBrazil

**Keywords:** * Alu-PCR*, breast cancer, gene insertions, gene deletions, invasive ductal carcinoma, recombination

## Abstract

*Alu*-PCR is a relatively simple technique that can be used to investigate genomic instability in cancer. This technique allows identification of the loss, gain or amplification of gene sequences based on the analysis of segments between two *Alu* elements coupled with quantitative and qualitative analyses of the profiles obtained from tumor samples, surgical margins and blood. In this work, we used *Alu*-PCR to identify gene alterations in ten patients with invasive ductal breast cancer. Several deletions and insertions were identified, indicating genomic instability in the tumor and adjacent normal tissue. Although not associated with specific genes, the alterations, which involved chromosomal bands 1p36.23, 1q41, 11q14.3, 13q14.2, occurred in areas of well-known genomic instability in breast and other types of cancer. These results indicate the potential usefulness of *Alu*-PCR in identifying altered gene sequences in breast cancer. However, caution is required in its application since the *Alu* primer can produce non-specific amplification.

## Introduction

Molecular genetic and cytogenetic analyses of breast cancer samples suggest that the development of this type of cancer involves the clustering of several, mainly structural, genetic alterations (Devilee and Cornelisse, 1994; El-Ashry and Lippmann, 1994; Beckmann *et al.*, 1997). Point mutations, such as small deletions and insertions, are the most widely described mutations, although genomic rearrangements are also very common (Montagna *et al.*, 2003; Belogianni *et al.*, 2004; Agata *et al.*, 2005). Chromosomal deletions and loss of heterozygosity (LOH) may indicate the inactivation of tumor suppressor genes in the affected region (Kenemans *et al.*, 2004).

One of the mechanisms proposed to explain the origin of deletions and insertions is based on the dispersion dynamics of transposable elements in the genome. According to Presneau *et al.* (1998), abortive integration of a transposon can be simultaneously responsible for a deletion and an insertion. The insertion of a transposon may damage DNA by interrupting the gene, but when unequal homolog recombination occurs several genes can be affected, with unpredictable consequences to the phenotype. Indeed, there is strong evidence of a relationship between transposable elements and human genetic diseases. For example, insertion of *Alu* elements appears to be involved in the etiology of 0.1-0.3% of human genetic diseases, including Tay-Sachs disease, Duchenne muscular dystrophy, complement deficiency, and breast, ovary and colorectal cancer (Batzer and Deininger, 2002; Chen *et al.*, 2005).

A relatively simple technique that has been used to investigate genomic instability in cancer is *Alu*-PCR (Tsongalis *et al.*, 1993; Furmaga *et al.*, 2003, 2004) that is based on the large number of copies of the *Alu* retroelement in the human genome. *Alu* elements are sequences of ~300 nucleotides (Cordaux *et al.*, 2006) known as SINEs (short interspersed nuclear elements), of which there are ~500,000 copies (Deininger and Batzer, 1999; Batzer and Deininger, 2002) that account for 10% of the human genome (Ng and Xue, 2006). Deletions or insertions between two elements are easily detected by PCR (Strout *et al.*, 1998; Suminaga *et al.*, 2000; Rowold and Herrera, 2000; Stenger *et al.*, 2001; Weichenrieder *et al.*, 2001). Since *Alu* elements can be inserted in opposite directions in a DNA sequence, it is possible to use only one primer in the PCR reaction to detect genetic alterations in cancer cells (Furmaga *et al.*, 2003). *Alu*-PCR does not search for a specific locus but yields a profile of bands of genomic DNA that may differ between tumor and normal adjacent (control) tissues of the same patient. The gain or loss of genomic material may involve large or small gene sequences associated with LOH and/or gene amplification.

According to Fumarga *et al.* (2003, 2004), *Alu*-PCR is a very sensitive technique that has the advantage of being able to detect novel genomic alterations without the need for prior knowledge of these sequences. *Alu*-PCR has been used to identify the genetic changes potentially involved in lung carcinoma metastasis (Furmaga *et al.*, 2003) and to distinguish typical pulmonary carcinoids from classic midgut carcinoids, which are histologically similar (Furmaga *et al.*, 2004). However, there is no information about the sequences of the altered bands that allows identifying the segments involved in the genetic gains or losses.

The aim of this study was to investigate the genomic instability of sporadic invasive ductal breast cancer by using *Alu*-PCR as an alternative approach to other methods commonly used to obtain comparative fingerprints of cancer and normal tissues, *e.g.*, AP-PCR (Peinado *et al.*, 1992), MS-AP-PCR (Gonzalgo *et al.*, 1997) and Inter-SSR-PCR (Basik *et al.*, 1997). Additionally, we sequenced the fragments involved in the gains or losses in order to identify their sequences and compared them with the human genome database.

**Figure 1:**
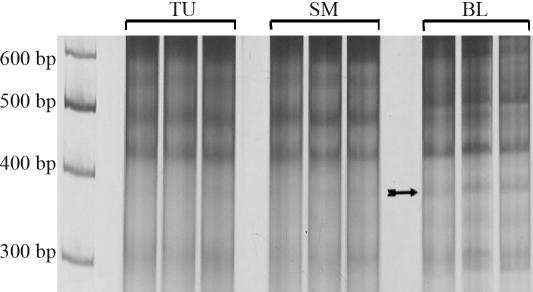
Electrophoretic profiles of three replicates of tumor (TU), surgical margin (SM) and blood (BL) DNA samples from patient no. 2 showing the reproducibility of the *Alu*-PCR profile in breast cancer. The arrow indicates a band present in the blood sample that is less visible in the surgical margin and absent in tumor tissue.

**Figure 2:**
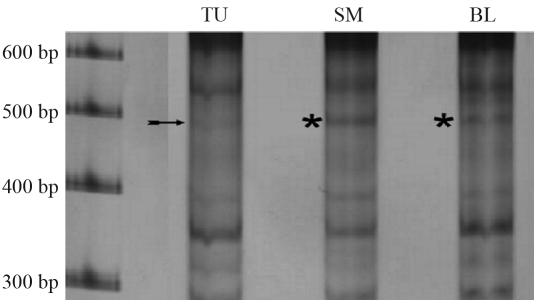
Electrophoretic profiles of tumor (TU), surgical margin (SM) and blood (BL) DNA samples from patient no. 5. Note the presence of two deletions in the tumor tissue, one of ~500 bp (sequenced from the corresponding band extracted from the blood sample, indicated by the asterisk) and another of ~600 bp (this band was fainter in the digitalized image).

**Figure 3:**
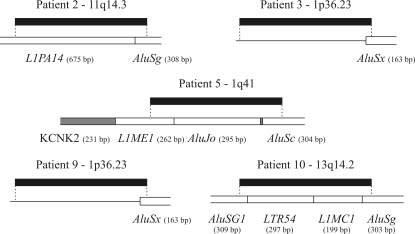
Schematic representation of the chromosomal position of each sequence extracted from the gels. The black boxes indicate the sequences that were analyzed, the empty boxes below each sequence represent transposable elements, and the gray boxes represent exons of the KCNK2 gene.

**Figure 4:**
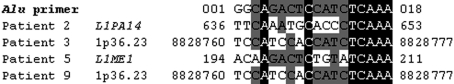
Alignment between the *Alu* primer used for the *Alu*-PCR technique and the LINE elements found in the bands of patients 2 and 5 and the flanking region without an *Alu* element found in patients 3 and 9.

## Material and Methods

### Samples

Genomic DNA was extracted from tumor and apparently normal tissues, surgical margins and blood of ten patients (50-69 years old) who underwent surgery for removal of invasive ductal breast carcinoma with grade II or III tumors. The samples were collected and the tumor grade was classified macroscopically by medical professionals of the Gynecological Oncology and Mastology Unit of the Department of Gynecology and Obstetrics, the Plastic Surgery Service of the Department of Surgery, and the Pathological Anatomy Service of the Department of Pathology and Forensic Medicine of the São José do Rio Preto School of Medicine. The material was donated after written informed consent from all participants and its use in this project was approved by the Ethics Committee at UNESP in São José do Rio Preto and by the National Committee for Ethics in Research (CONEP, registration no. 10811).

### Genomic DNA extraction

Genomic DNA was extracted from fresh solid tissue by using the DNA extraction protocol described by Sambrook *et al.* (1989).

### Alu-PCR

Hot-start PCR was done with 150 ng of genomic DNA in a reaction with a final volume of 25 μL containing 1.25 U of *Taq*Bead hot-start polymerase (Promega), 200 μM of each dNTP, 1X PCR buffer, 1.5 mM MgCl_2_ and 100 ng of *Alu* initiator (5'-GGCAGACTCCATCTCAAA-3') that anneals at the 3' end of the *Alu* element, immediately before the poly-A tail. The cycling parameters were: initial denaturation at 94 °C for 3 min, followed by 40 cycles of denaturation at 94 °C for 2 min, annealing at 55 °C for 2 min and extension at 72 °C for 3 min. The final extension was at 72 °C for 10 min. The amplification products were separated by electrophoresis in 7.5% polyacrylamide gels and stained with silver nitrate (Caetano-Anollés and Bassam, 1993). Initially, the gels were run at a constant power of 300 V for 30 min followed by 4 h at a constant power of 100 V at room temperature. The gels were fixed using a standard procedure and dried on cellophane and 20% glycerol, as described by Ceron *et al.* (1992).

### Selection of candidate bands and extraction

The bands representing possible insertions, deletions and amplifications were extracted from the dried gels and eluted overnight at 37 °C in elution buffer. The eluted material was then centrifuged and the DNA was precipitated, dehydrated and eluted in 10 μL of elution buffer followed by storage at -20 °C for subsequent cloning and sequencing.

Quantitative (gain or loss of a specific band) and qualitative (change in band intensity) analyses of the *Alu* profile were done by comparing the profiles of 100-700 bp fragments obtained with this technique since bands in this size range provided better staining and visualization. The bands were visualized with a UV lightbox since this allowed the detection of weakly stained bands that were difficult to see in digitalized images.

### Cloning and sequencing

Fragments extracted from the polyacrylamide gels were amplified under the same conditions as the *Alu*-PCR. The products were separated on 1% agarose gels, from which they were subsequently extracted, purified and cloned. Two colonies in which the presence of the plasmid with an insert was confirmed were selected and the plasmids were extracted using the alkaline lysis “miniprep” method (FlexiPrep kit, Amersham), according to the manufacturer's instructions. Sequencing was done in an ABI 377 sequencer and sequence consensus was determined with the BioEdit Sequence Alignment Editor program (Hall, 1999). Sequence identification and chromosomal localization were determined by searching the human genome database with the basic local alignment tools BLAST and BLAT.

## Results

Differences in the electrophoretic band profile were observed in 9 out of the 10 cases studied. The total number of quantitative and qualitative alterations observed was 3.30 ± 2.98 per patient (mean ± SD Table 1), and the most frequently observed alteration was the loss of bands in tumor tissue (1.00 ± 1.05 alterations per patient). Two figures are provided to illustrate the results obtained. Figure 1 shows the electrophoretic profiles of the three replicates of tumor, surgical margin and blood DNA samples from patient n. 2 and confirms the reproducibility of the results. This figure also shows that blood contained a band that was absent for the surgical margin and tumor samples. Figure 2 shows the electrophoretic profiles of the tumor, surgical margin and blood DNA of patient no. 5. In this case, the tumor tissue showed two deletions, one of ~500 bp (for which the corresponding band in blood was sequenced) and another of ~600 bp that was only weakly visible in the digitalized image.

Five bands from different cases were selected for cloning and sequencing. Table 2 summarizes the tissues (tumor, surgical margin or blood) that were altered in five patients and shows the band that was selected for sequencing, the type of genetic alteration (gain or loss of a sequence), the band size, the gene location of each altered sequence and the identity of each clone with a sequence in the human genome. The chromosomal location of these sequences is shown in Figure 3. All of the sequences had a transposable element at at least one extremity or internally, within the sequence. The only case with *Alu* sequences at both extremities, as shown by *Alu*-PCR, was patient 10: the sequence had an *AluSg1* sequence at the 5' end and an *AluSg* sequence at the 3' end. In patient no. 2, the sequence consisted of an *L1PA14* element of the LINE superfamily, followed by an *AluSg* sequence at the 3' end. In patient no. 5, the sequence that was amplified belonged to an intronic region of the KCNK2 gene that harbored three transposable elements, *L1ME1*, *AluJo* and *AluSc*, of which *AluJo* was possibly a complete element because of its 294 bp size. Patients 3 and 9 had exactly the same altered region, with an *Alu* element at the 3' end and no other transposable elements at the 5' end. Although they shared the same altered region, patient 3 presented a sequence gain and patient 9 a sequence loss.

Figure 4 shows the non-specific alignments between the *Alu* primer and the LINE elements found in the sequenced fragment of patients 2 (*L1PA14*) and 5 (*L1ME1*) and in the flanking region without an *Alu* element in patients 3 and 9. As can be seen, 10-13 of the 18 nucleotides of the *Alu* primer occurred in the 3' end of the *L1PA14* and *L1ME1* elements or in the intergenic flanking region. The high similarity (55%-72%) between the sequences of these regions and the *Alu* primer apparently accounted for the non-specific amplifications.

## Discussion

Genomic instability accompanies the progression of neoplasia and probably predisposes the individual to additional genetic alterations that confer proliferative advantages to the cells. The sequence between two *Alu* elements may be more susceptible to deletions and unequal recombinations and, consequently, to the formation of new rearrangements. This hypothesis is supported by Batzer and Deininger (2002) who stated that the high density of *Alu* elements in human DNA provides “hot spots” for homolog recombination and chromosomal translocation.

In the present work, genomic DNA from tumor tissue, surgical margins and blood from ten patients who underwent surgery for the removal of invasive ductal breast carcinoma was analyzed with *Alu*-PCR. The most frequently observed alteration in tumor tissue was the loss of fragments, with an average loss of one band for every 3.3 altered bands. This value was approximately twice that of the gain of new bands, on average 0.6 per patient for the same tissue. Surgical margins also showed alterations in the *Alu*-PCR profile, with frequencies of 0.5 ± 0.97 new bands and 0.5 ± 0.85 lost bands per patient This result may reflect the presence of tumor cells that are not detectable by the routine histopathological examination of surgical margins (Cesar *et al.*, 2004, 2006; Hughes *et al.*, 2006). Similarly, qualitative alterations in tumor tissue may be caused by the presence of normal stroma cells or leukocytes.

The sequences corresponding to the altered bands (1p36.23, 1q41, 11q14.3 and 13q14.2) belonged to regions associated with genomic instability and cancer. The loss of genetic material in chromosome 1p has been observed in many types of cancer, and is particularly frequent in breast, lung, endometrium and ovary cancer and in gliomas (Barbashina *et al.*, 2005). The loss of heterozygosity suggested the presence of one (Ragnarsson *et al.*, 1999) or several (Barbashina *et al.*, 2005) tumor suppressor genes in this chromosomal arm.

Studies of different human neoplasias, including breast cancer, teratoma, astrocyte glioma, osteosarcoma, hepatocellular carcinoma and prostate cancer, have suggested the presence of a tumor suppressor gene in the long arm of chromosome 1 (Ding, 1992; Mertens, 1993; Murty *et al.*, 1994; Li *et al.*, 1995; Loupart *et al.*, 1995; Berthon *et al.*, 1998). In BLAST analysis, the sequence at 1q41 was located in an intronic region of the KCNK2 gene which coded for a member of the two-pore-domain background potassium channel protein family. Another member of this family, TASK3 or KCNK9, was also amplified and showed elevated expression in breast tumors. Elevated expression of TASK3 in cell lines confers resistance to hypoxia and serum deprivation, suggesting an important physiological role for this gene in breast tumorigenesis (Mu *et al.*, 2003). The loss of 500 bp by the KCNK2 gene deserves further studies since intron alterations can affect internal promoter or splicing sites, thereby changing gene expression.

A loss of heterozygosity in the 11q14.3 region has been observed in head and neck cancer and correlated with tumor grade (Glavac *et al.*, 2003). In breast cancer cell lines studied by comparative genomic hybridization (CGH) and spectral karyotyping (SKY) the regions most commonly affected by LOH included 11q14-qter (Kytola *et al.*, 2000). The region 13q14 contains genes related to a variety of neoplasias, such as RB1, which however is located in a segment distant from the sequence analyzed. Some expressed sequence tags (DB448514, CD359283 and DB445670) have been mapped to the segment that comprises the sequenced clone, and the nearest gene is LCP1 of the plastin family of actin-bundling proteins. Foran *et al.* (2006) observed elevated expression of L-plastin associated with increased proliferation and invasion and loss of E-cadherin in a colorectal cancer cell line and suggested that this protein played an important role in metastasis.

*Alu*-PCR identified structural genetic alterations such as deletions and insertions and provided a profile of quantitative and qualitative changes in the samples studied here. These rearrangements were expected to be flanked by two *Alu* elements. The sequences showed three patterns in the five patients. The expected pattern was observed only in patient 10, with *Alu*Sg1 and *Alu*Sg flanking the insertion in tumor and marginal tissues. On the other hand, patients 2, 3, 5 and 9 had *Alu* at the 3' end but not at the 5' end. Patients 2 and 5 harbored the retroposon L1 (*L1PA14* and *L1ME1*). L1 elements are long interspersed nuclear elements (LINES) associated with insertion mutations and chromosomal rearrangements that have been correlated with several human diseases, including breast cancer (Ostertag and Kazazian Jr, 2001). Patients 3 and 9 shared the same altered sequence with no transposable element at the 5' end. The amplification of these non-*Alu* sequences probably resulted from the lack of absolute specificity of the *Alu* primer at its 3' end. Non-specific annealing consistently occurred at the 5' end of the fragment, but we have no explanation for this selectivity.

*Alu*-PCR was originally designed to identify rearrangements mediated by *Alu*s. However, as shown here, the *Alu*-primer amplified other sequences in addition to those flanked by *Alu*s. Since L1 is a major source of non-specific genetic instability in humans the amplification also reveals genetic instability and do not compromise the efficiency of the technique. Our findings show that caution must be exercised when using this technique because of the risk of obtaining a spurious amplification, as observed for the 1p36.23 region. Nevertheless, these spuriously amplified sequences are interesting because they may be involved in genetic rearrangements that include a gain or loss of fragments of DNA. Thus, *Alu*-PCR can be helpful as a preliminary strategy for screening regions of genomic instability involved in the initiation and progression of cancer and other diseases. The identification of unstable segments can function as potential biomarkers for the early detection of tumors and may be of prognostic use in monitoring disease progression.

## Figures and Tables

**Table 1:** Quantitative and qualitative changes observed in the *Alu* profile.

	Quantitative								Qualitative			Total
Sample	Band gain				Band loss							
	TU	SM	BL		TU	SM	BL		TU	SM	BL	
Patient 1	1	1	0		0	0	0		0	0	0	2
Patient 2	0	0	0		3	2	0		0	0	0	5
Patient 3	3	3	0		2	0	0		1	0	0	9
Patient 4	0	1	0		1	1	0		2	1	0	6
Patient 5	1	0	0		2	2	0		1	0	0	6
Patient 6	0	0	0		1	0	0		0	0	0	1
Patient 7	0	0	0		0	0	0		0	0	0	0
Patient 8	0	0	0		1	0	0		0	0	0	1
Patient 9	0	0	0		0	0	0		1	0	0	1
Patient 10	0	0	0		0	0	0		1	1	0	2
Mean ± SD	0.50 ± 0.97	0.50 ± 0.97	0		1.00 ± 1.05	0.50 ± 0.85	0		0.60 ± 0.69	0.20 ± 0.42	0	3.30 ± 2.98

BL – blood, SM – surgical margin, TU – tumor.

**Table 2:** Sequencing results for the candidate bands.

Patient	Altered tissue	Band alteration	Tissue analyzed	Band size	Genomic localization (position)	Identity (%)
2	TU and SM	Loss	BL	300-400 bp	11q14.3 (89867448-89868025)	98.7
2	TU and SM	Loss	BL	300-400 bp	11q14.3 (89867448-89868009)	99.3

3	TU and SM	Gain	TU and SM	400-500 bp	1p36.23 (8828760-8829352)	96.8
3	TU and SM	Gain	TU and SM	400-500 bp	1p36.23 (8828760-8829328)	98.0

5	TU	Loss	BL and SM	500 bp	1q41 (213251146-213251604)	99.8
5	TU	Loss	BL and SM	500 bp	1q41 (213251146-213251604)	99.8

9	TU and SM	Loss	BL	300-400 bp	1p36.23 (8828760-8829350)	98.6
9	TU and SM	Loss	BL	300-400 bp	1p36.23 (8828760-8829354)	98.7

10	TU and SM	Gain	TU and SM	500 bp	13q14.2 (47667917-47668511)	98.9
10	TU and SM	Gain	TU and SM	500 bp	13q14.2 (47667917-47668496)	99.1

BL – blood, SM – surgical margin, TU – tumor.
